# Draft Genome Sequence of the Luminescent Strain *Vibrio campbellii* LB102, Isolated from a Black Tiger Shrimp (*Penaeus monodon*) Broodstock Rearing System

**DOI:** 10.1128/genomeA.00342-17

**Published:** 2017-05-18

**Authors:** Sujeet Kumar, Ashok Kumar Jangam, V. Akhil, Vidya Rajendran, Vinaya Kumar Katneni, J. Joseph Sahaya Rajan, Monendra Grover, Viswas Konasagara Nagaleekar, S. V. Alavandi, K. K. Vijayan

**Affiliations:** a ICAR-Central Institute of Brackishwater Aquaculture, Chennai, India; b Centre for Agricultural Bioinformatics, ICAR-Indian Agricultural Statistics Research Institute, New Delhi, India; c ICAR-Indian Veterinary Research Institute, Izatnagar, Bareilly, India

## Abstract

We report here the genome sequence of *Vibrio campbellii* LB102, isolated from the broodstock rearing system of a shrimp hatchery in India. Sequence analysis revealed the presence of effector toxins of the type III (YopT, sharing 39% identity with *Yersinia pestis*) and type VI (VgrG-3 and hemolysin coregulated protein of *V. cholerae*) secretion systems.

## GENOME ANNOUNCEMENT

*Vibrio campbellii* is an emerging bacterial pathogen that affects the mysis and early postlarval stages of penaeid shrimp. It closely resembles the marine pathogen *V. harveyi* and is almost indistinguishable from it based on phenotypic characteristics, 16S rRNA gene sequence analysis, and DNA-DNA homology (1). In the past, *V. campbellii* was often misidentified and classified as *V. harveyi* (2). Development of multilocus sequence typing using housekeeping genes and whole-genome sequencing, however, have paved the way for their differentiation (2). While investigating luminescent bacterial disease, *V. campbellii* LB102 was isolated in 2006 from a broodstock rearing system at a tiger shrimp hatchery on the southeast coast of India (3). The strain was luminescent, was confirmed as *V. campbellii* using *rpoD* gene sequence analysis, and was found to be virulent in an experimental challenge trial on postlarvae.

The genomic DNA from LB102 was extracted using a Qiagen genomic DNA extraction kit. The 350-bp genomic library was sequenced using the Illumina HiSeq2500 platform, generating 59,844,642 paired-end reads (100 bp) with a coverage of 997×. The reads were assembled *de novo* using SPAdes version 3.10.0 (*k*-mer = 55) (4). The assembly contained 90 scaffolds (107 contigs) of ≥200 bp, covering 5,588,138 bp. The size of the genome was 5.59 Mb with a 45.5% G+C content. The three scaffolds accounted for 50% of the assembly (*L*_50_), with the largest scaffold size being 1,047,310 bp. Average nucleotide identity of the LB102 whole genome was calculated using Pyani (5), a Python module, and found to be 97.9% for *V. campbellii* LMB29 strain. Gene prediction and annotation were performed using the Rapid Annotations using Subsystems Technology (RAST) server (6) and the NCBI Prokaryotic Genome Annotation Pipeline (7). The LB102 genome had total 5198 genes; 4920 protein coding, 15 rRNA, 121 tRNA, 4 other RNA and 138 pseudogenes.

The LB102 genome contained RTX toxin (cytolysin), vibriolysin (extracellular zinc protease), aerolysin (pore forming toxin), SpvB (Actin-ADP-ribosyltransferase), thermolabile hemolysin, chitinase, zinc metalloprotease, alkaline serine protease, phospholipase C, DNase, and collagenase. Secretion systems play a crucial role in the virulence of bacterial pathogens. The genome of *V. campbellii* LB102 had secretion systems of types I, II, III, and VI. Four genes encoding homologs of *V. cholerae* toxins—VgrG-3, hemolysin coregulated protein (hcp), transcriptional activator ToxR, and transmembrane regulatory protein ToxS—were present in the genome. Both VgrG-3 and hcp are effector proteins secreted by a type VI secretion system in *V. cholerae* (8). The genome also contained an effector protein of the type III secretion system, YopT, which is homologous to the effector protein of *Yersinia pestis* (9). YopT is known to cause cytotoxicity through the depolymerization of actin filaments (9). Sequence data showed the presence of various colonization factors, such as mannose-sensitive hemagglutinin (MSHA), type IV pilin (PilA), V10 pilin, and fimbrial protein, in LB102 genome. In most of the Gram-negative bacterial pathogens, type III and type VI secretion systems are known to play major roles in their pathogenesis. Further investigation is required to understand the role of these putative effector proteins and colonization factors to understand the virulence mechanism of *V. campbellii* in shrimp.

### Accession number(s).

This whole-genome shotgun project has been deposited at DDBJ/ENA/GenBank under the accession number MWRX00000000. The version described in this paper is the first version, MWRX01000000.
